# The Necessity of Appointing Professed Dentists to All Public Hospitals and Dispensaries

**Published:** 1844-12

**Authors:** 


### Collectanea.

***The Necessity of appointing Professed Dentists to all public Hospitals and Dispensaries.***—It has become so completely the custom for the *paying* orders of the metropolis to employ professed dentists in all that relates to the teeth, that surgeons, properly so called, have ceased to take any interest in, or pay any attention to the subject, and are in such a felicitous state of ignorance, that the lancing a gum or extracting a tooth is, generally speaking, the extent of their accomplishments in *dental* surgery; nor do they even in the latter simple operation rival our old friend of dramatic memory, Crushjaws of Carshalton, who "extracts a tooth with perfect *pleasure* to the patient." Still there is a difference in *their* mode of operating, the head feeling in one case as if crushed by a broad-wheeled wagon, in another, merely run over by a cab.

But diseases of the teeth are not confined either to the *paying* orders or to the metropolis and large provincial towns, where dentists are more plentiful than blackberries in their suburbs, but occasionally affect inhabitants of the country, where they of necessity come under the care of the general practitioner, and the *poor* of the metropolis, who must either go to a parish surgeon who knows little of the subject, or to a hospital, where the students (to whom these cases are turned over) know less.

A *pure* surgeon, or a fashionable apothecary, can scarcely be expected to pay any attention to a subject of such minor importance as the teeth, or soil their aristocratic fingers by touching a key instrument or a pair of tooth forceps; but with country practitioners the case is different. Tooth drawing, and other operations of dental surgery, form an important branch of practice, and their judgment is frequently taxed to regulate the teeth and perform all the duties of the dentist.

Surely, then, it should be placed in the power of every medical student to acquire a knowledge of dental surgery with as much facility as possible, without any unnecessary expenditure of time or trouble: and as the hospital surgeons seem to think this branch of the profession beneath *their dignity*, a professed dentist should be attached to every hospital and dispensary, and no *pupil* should be allowed to operate but under his sanction; for though there is a tacitly-understood compact that those who accept the benefits of a hospital are used as media of instruction, there is neither justice nor humanity in making them mere blocks for experiments of untutored clumsiness.

As matters are managed at present, no one who could raise a shilling would take *advantage* of hospital dentism, or submit his jaws to the tender mercies of its pupils, much less consult them in any case requiring knowledge or judgment; for though these young gentlemen have been in the *habit* of performing the *minor* operations, know as much as they probably ever will of the subject, and are fair examples of dental surgery as practised by surgeons, they are seldom so expert as to induce any one to trust them a second time.

We have more than once felt for a poor *patient*, as we have seen him place his head back in the operating chair in all the confidence of blessed ignorance, and witness the process of student tooth-drawing, in which it seemed a doubt which would give way first—the tooth, the jaw, or the instrument—and which ended either in a handsome piece of the alveolar process (by the ignorant supposed to be the jaw itself) being brought away with the tooth, or by the tooth itself being extracted without the fangs. Occasionally we have seen the tooth broken short off, and the operator precipitated on to his nose by the violence of the reaction. In one case the wrong tooth is taken out; in another its neighbor thoroughly loosened; and now and then, by a clever arrangement of the instrument, the operation is made a double one, and *two* teeth deducted at the same time. Whatever the result of the operation to the *patient*, it is always accompanied by considerable exertion to the operator, who turns round to the witnesses of his labours, with, "What a tremendous tooth!" (including the alveolar process, which he probably considers part of it,) or "How devilish tight in!" or some equally scientific observation.

This is not as it should be: the poor are entitled to the best advice and most expert assistance a hospital can *be made* to afford, and the students have a right to every opportunity of learning their profession; while as matters are managed at present, neither the one intention nor the other is answered, and hospital assistance (as far as dental surgery is concerned) is not a boon to the patient, and anything but an advantage to the student.

It may be thought that the poor have no right to expect the services of a professed dentist, and that the rough hands of a student, reeking from the dissecting-room, are better adapted to their mouths. It may be; but surely they ought not to be exposed to unnecessary torture, and this can be prevented only by the appointment of professed dentists *to all hospitals and dispensaries,* and by these duties being properly defined and performed—an arrangement that would be not only valuable to the patients, but doubly so to the pupils; who, whatever rank they may afterwards hold in the profession—but more particularly if they practice in the country—will find the being good tooth drawers no mean addition to their other qualifications. At present, so much is dentism at a discount, (except as a separate profession,) that not one in twenty of the students, who has gone through his other studies with credit, knows more on the subject of the teeth than that they are shed at *some* period of life, and are totally incompetent to perform any of these operations (properly) except perhaps the very simple one of lancing the gums.

We remember a little boy of about nine years old, who had a wide gap where the front teeth should have been, being brought to our surgery. As he was beginning to shed the *double* teeth, we thought it strange that all the incisors should not have made their appearance, particularly as the three he had, seemed to be full grown. On inquiry, however, we found that he had been a victim to hospital dentism. His mother, who was a poor woman, finding the *second* teeth growing out *before* the others, and beginning to cut his lip, sent him, in the care of an elder sister, to M—— Hospital, where a young gentleman lugged out the second teeth that *appeared most in the way* and left the others, which in a short time came out without his assistance. Of course the injury was irreparable.

Why is there not another professor of dental surgery appointed at King's College Hospital since Mr. Tomes seceded? Has it never struck the directors of that establishment, that they are bound to afford *proper* relief to the poor, and the means of a *complete* medical education to the students? Are the teeth of no importance in the animal economy? we have always been foolish enough to imagine, that articulation depends in some measure on their aid, and that the important process of digestion is little likely to be properly performed if the teeth are not in *working* order, at least. Oh, but there are dentists! So there are oculists and aurists; but these gentlemen do not practice in country villages, or even small towns; nor is the army or the navy provided with them; yet none are exempt from diseases of the teeth and gums: and though it is unnecessary that a surgeon should be able to manufacture artificial teeth, he should be acquainted with all their diseases, and their most successful mode of treatment, when a professed dentist is not at hand. How are the students of King's College Hospital to acquire a knowledge of all this? By instinct? Or is it that the *professors* turn up their honorable noses at a dentist coadjutor? or are they afraid that their own shining talents may be thrown into the shade, by the appointment of a man who knows *too much?*

That the Charing Cross Hospital should be in the same predicament is not so wonderful, for the *enormous* opportunities of other kinds the pupils of that establishment have of acquiring a knowledge of their profession, more than compensate so trifling an omission. If the students do not know how to draw a tooth, they will probably be able to *perform,* bungle through, *some how or other,* an operation for the stone, and that will of course be equally satisfactory to their tooth patients. But the London, and North London Hospitals, both situated in poor localities, where the common operations of dental surgery are in constant requisition, ought not to be supported, unless a professor be appointed. Both the poor and the students have a claim to the advantage, for we know of no situation in which diseases of the teeth are more rife, or where the services of a scientific dentist are more required.

It is of no use, however, to *appoint* professors of dental surgery, unless they are *properly* appointed. They should not be merely the nominees of one or more of the medical officers, most of whom have some *pet dentist,* but should obtain their appointments by fair and open competition, assisted, but not dictated to, by these gentlemen. From what we know of these fraternizations, we will venture to say, that fine turbot and superior champagne have more influence in these appointments than any paltry considerations of professional competence.

We recollect a Scotchman, who could scarcely read or write, (but perhaps these may be considered unnecessary qualifications,) being appointed to one of the largest hospitals. No doubt he was duly qualified, as a few months previously he was employed as a *blacksmith* at Braithwaites', the engineers, an admirable preparation for his professorship! His professional qualifications, however, had nothing to do with the matter, for his appointment was the result of liberally feasting the directing surgeons and medical staff of the hospital (countrymen of course) with champagne, calf's head, and *real* turtle, (mock turtle would have been too cannibalish.) We shall shortly publish an account of this infamous job.

The appointment of dentists to the Middlesex, Westminster, and St. Thomas' Hospitals were not the acts of the subscribers, but of the medical officers under the rose: and although the gentlemen are duly qualified, and highly respectable, we detest a system by which men are appointed to public situations, because they have tickled the palatian nerves of the medical men of the establishment with the good things of this life, or by that tickle-me-tickle-you-arrangement. Ask Mr. A, the *pure,* and he will tell you that Mr. B is the only scientific (that is the word) dentist in London, while Mr. B will return the compliment, by assuring you that Mr. A is the *first* surgeon.

No gentleman should fill the professorship of dental surgery, whose practice is so large that he must sacrifice either *that* or the hospital to which he is attached, (common sense teaches which it will be,) and who accepts the appointment only for the reputation of being connected with a public charity. To be of use to the patients, or to the students, he should attend twice or three times a week, or make such an arrangement that, in any particular case, both the patients and the pupils may attend at his own house.

Little good will be done by a bald and parrotted course of lectures: to be of use, they must be of the most practical kind, and accompanied by the results of his practice, successful or unsuccessful, both public and private. He must not take it for granted that his pupils understand the commonest details of the subject, but must begin at the beginning, *correcting any bad habits* his pupils may have acquired, and exemplifying the commonest operations. Was this plan carried out to the fullest extent, we should not meet with the mistakes in dental surgery that now disgrace the profession. Pulling a dens sapientia would not be mistaken for a first and second molar tooth; the contour of mouths would not be spoiled by the improper removal of the deciduous teeth; the operation of extracting would be performed less frequently and less painfully; and those dreadful accidents which now too frequently disgrace our public hospitals and dispensaries, to which no professed dentist is attached, would be avoided.—*Forceps.*

